# Anaerobic fermentation of soybean meal by *Bacillus subtilis* ED-3-7 and its effect on the intestinal microbial community of chicken

**DOI:** 10.1016/j.psj.2024.104564

**Published:** 2024-11-27

**Authors:** Wei Liu, Wei Wang, Jia Li, Hongya Li, Tongguo Gao, Baocheng Zhu

**Affiliations:** a College of Life Sciences, Hebei Agricultural University, Baoding, China; b Feed Microbial Technology Innovation Center of Hebei Province, Baoding, China

**Keywords:** *Bacillus*, Soybean meal, Bacterial community structure, Solid-state fermentation

## Abstract

A strain named ED-3-7 with a high protease-producing ability was screened in a previous study. This strain can be used for the anaerobic fermentation of soybean meal (SBM) to degrade macromolecular antigen proteins and antinutritional factors. We here evaluated the nutritional quality of the anaerobic fermented SBM and its effects on the chicken intestinal microbial community. Crude protein and acid-soluble protein contents increased by 11.68% and 342.61%, glycinin and β-conglycinin decreased by 82.04% and 88.42%, urease content decreased by 90.10%, and the trypsin inhibitor content was lower than the range specified in the detection kit. After being fed with the fermented SBM, the average daily gain, nutrient digestibility of the chickens increased, and their intestinal bacterial community exhibited significant changes. The richness and diversity of bacterial species decreased, and *Lactobacillus* became the dominant genus, which was conducive to the health of chicken intestines. The experimental results revealed that ED-3-7 anaerobic fermentation improved the nutritional quality of SBM and had beneficial effects on chicken intestines. Thus, the strain could be used for large-scale industrial production.

## Introduction

Proteins are a component in animal feed that helps to meet the nutritional requirements of animals. The protein content of the raw material is among the crucial criteria for selecting animal feed. Soybean meal (SBM) is the most frequently used dietary protein in poultry farming (Guo et al., 2020). It is widely used in poultry farming for its high protein content, balanced amino acid composition, and suitable price (Qi et al., 2023). However, SBM has various antinutritional factors (ANFs) such as antigenic proteins, phytic acid, and trypsin inhibitors (TIs) (Huang et al., 2023), that inhibit nutrient absorption by chicks, ultimately resulting in gastrointestinal dysfunction. In severe cases, gastrointestinal dysfunction can cause water loss and diarrhea, leading to animal death and loss of economic benefits (Li et al., 2020). Therefore, the content of ANFs and macromolecular protein in SBM must be reduced in the feeding of chicks, and improve the absorption of nutrients in chicks.

Microbial SBM fermentation is among the commonly used methods for reducing ANFs and improving the SBM nutritional quality. After SBM fermentation, ANFs are reduced by 70%-90% (Li et al., 2019b), and the macromolecular protein in SBM is degraded into small molecular substances such as peptides and amino acids. This eases the digestion burden on animal intestines and stomachs, improves digestion and absorption capacities, and reduces nitrogen emission into the environment (Shi et al., 2017a; Yuan et al., 2017). Additionally, beneficial microorganisms in the fermented SBM could improve the intestinal environment of chickens and promote their intestinal digestion and absorption (Jazi et al., 2018).

*Bacillus* is among the most commonly used strains in SBM fermentation. After aerobic fermentation by *Bacillus* sp., the contents of the ANFs glycinin and β-conglycinin reduced by 77.9% and 57.1%, and the macromolecular antigen proteins were almost completely degraded (Huang *et al.*, 2023). Most current studies focus on the *Bacillus*-mediated aerobic fermentation of SBM. The raw materials used for aerobic fermentation must be sterilized and consume a lot of energy. Therefore, the study of anaerobic fermented SBM can be conducted according to the characteristics of the *Bacillus* facultative anaerobic organism. After anaerobic fermentation, the degradation level of ANFs and macromolecule proteins can reach the level after aerobic fermentation (Zheng et al., 2017). Furthermore, the fermented material requires no sterilization, which saves energy and cost (Yang et al., 2020). Anaerobic fermentation can replace aerobic fermentation for the study of the reduction of ANFs and macromolecular proteins in SBM. The fermented SBM can improve the chickens’ intestinal flora and exert a favorable effect on their intestinal health (Jazi *et al.*, 2018; Ovissipour et al., 2009). However, the effect of FSBM prepared through anaerobic fermentation on the microbial community structure in the intestinal tract of chickens has not been reported.

In our previous work, we screened a strain called ED-3-7 (Yao et al., 2021). This strain exhibited high acid protease- and neutral protease-producing capabilities. This study evaluated the effect of this strain on anaerobic fermentation and the effect of FSBM on the growth and intestinal microbiota of chickens.

## Materials and methods

### Strain and reagent

ED-3-7 is a strain with high acid and neutral protease activities. After culturing in nutrient broth medium (5 g/L peptone, 3 g/L of beef extract, 5 g/L of NaCl, 1 L of distilled water, pH 7.0-7.2) at 37°C with shaking at 180 rpm for 24 h, the amount of acid and neutral proteases formed were 47.85 and 283.17 U/mL, respectively (Yao *et al.*, 2021).

Defatted SBM was purchased from Shandong Koufu Oils & Grains Co., Ltd. (Binzhou, China). All reagents, except molecular biology-related reagents, were analytically pure and purchased from local reagent companies.

### SBM solid-state fermentation

ED-3-7 cells were cultured in a nutrient broth medium at 37°C with shaking at 180 rpm for 48 h. The unsterilized SBM was used in anaerobic fermentation. Then, 10^9^CFU/kg of microbial culture was inoculated into the unsterilized solid-state fermentation (SSF) medium (SBM 88.0%, corn flour 10.0%, (NH_4_)_2_SO_4_ 2.0%); mixed with the same amount of water; placed in a barrel (6 kg); compacted and sealed; and fermented at room temperature for 14 days.

### Determination of FSBM chemical composition

The crude protein was detected using the automatic Kjeldahl apparatus (K9840, Haineng, China). The content of trichloroacetic acid-soluble protein (TCA-SP) was determined using the methods of Ovissipour et al. (2008). Glycinin, β-conglycinin, and TI were analyzed using a commercially available ELISA assay kit (Longkefangzhou Bio-Engineering Technology Company, Beijing, China). The phytic acid content was determined using the ferric chloride colorimetric method (McKie and MccleAry, 2016). The total urease activity was determined according to the Chinese National Standard GB/T 8622-2006 (Determination of urease activity in soyabean products for feeds). The contents of neutral detergent (NDF), acid detergent fiber (ADF), and ash, and the pH were determined according to the methods of Van Soest et al.,(1991).

### Strain identification

The complete ED-3-7 genome was extracted using the Easypure Bacteria DNA kit (Beijing TransGen Biotech Co. Ltd.). The universal primers were used for PCR amplification, as the forward primer was 27F: 5′-GAGAGTTTGATCCTGGCTCAG-3′, and the reverse primer was 1495R: 5′-CTACGGCTACCTTGTTACGA-3′. The base fragments were located at positions 27–46 and 1476–1495 of the 16S rRNA gene, respectively (subject to the base position of the 16S rRNA gene of *Escherichia coli*). The reaction mixture for PCR contained 1 μL template DNA, 1.6 μL of 25 mmol/L dNTP mixture, 1 μL of 10 μmol/L 27 F, 1 μL of 10 μmol/L 1495 R, 2 μL of 10 × ExTaq buffer (containing Mg^2 +^), and 0.2 μL ExTaq DNA polymerase. Then, ddH_2_O was added to make the volume 20 μL. The PCR conditions were as follows: 95°C for 5 min; 95°C for 1 min, 56°C for 1 min, and 72°C for 1 min 30 s; and 30 cycles of 72°C for 10 min. The amplification products were detected through 1% agarose gel electrophoresis and sequenced by Beijing Liuhe Huada Gene Technology Co., Ltd) (Cheng et al., 2017). The physiological and biochemical characteristics of strain ED-3-7 were identified according to Bergey's Manual of Systematic Bacteriology (Garrity, 2007).

### Chicken feeding

This study involving live animals met the guidelines of the Institutional Animal Care and Use Committee (IACUC). The animal use protocol was approved by the Institutional Animal Care and Use Committee of Hebei Agricultural University. All data were collected by adhering strictly to the requirements of this Committee. All test methods are designed and operated according to ARRIVE guidelines. Utmost efforts were taken to minimize the number of animals used with little or no suffering.

In total, 180 White Feather Broilers (7 days age) were selected for a 2-week feeding experiment and randomly assigned to 6 pens, 30 animals for each pen. The 6 pens were divided into two groups: SBM and FSBM, with 3 replicates each. By referring to the *Nutrient Requirements of Poultry: Ninth Revised Edition* (Dale, 1994) and China's Feeding Standard of Chicken (NY/T33-2004, China), the basal diet was made of corn and SBM (Table 1). In the FSBM group, the birds were fed the diet in which SBM was replaced with FSBM.

**Table 1:** composition and nutrient level of experimental diets (air-dry basis, %).

Items	SBM	FSBM	Items	SBM	FSBM
Ingredient			Calculated nutrient level		
Corn	54.20	55	AEM (MJ/kg)	12.54	12.54
Soybean meal	33.62	-	Crude protein	21.50	21.50
Fermentation Soybean meal	-	33.62	Calcium	1.00	1.00
Wheat bran	0.20	2.80	Total phosphorus	0.76	0.78
Fish meal	4.00	0.40	Available phosphorus	0.45	0.45
Stone powder	1.55	1.30	Lysine	1.15	1.15
CaHPO_4_	1.30	1.72	Methionine	0.56	0.57
Soybean oil	3.98	4.12	Methionine+Cystine	0.91	0.91
NaCl	0.20	0.20			
Lysine	0.23	0.06			
Methionine	0.22	0.28			
Premix#	0.50	0.5			
Total	100	100			

# The premix provided the following per kilogram of complete feed: vitamin A 8,000 IU, vitamin D3 1,000 IU, vitamin E 20 IU, vitamin K 0.5 mg, vitamin B1 2 mg, vitamin B2 8 mg, vitamin B6 6 mg, vitamin B12 0.03 mg, niacin 35 mg, D-pantothenic acid 10 mg, folic acid 0.55 mg, biotin 0.18 mg, Fe 100 mg, Cu 8 mg, Zn 100 mg, Mn 120 mg, I 0.7 mg, Se 0.3 mg.

### Growth performance of broiler chicken

The body weight (BW) per replicate of the chickens was recorded at 7 and 21 days to calculate the average daily gain (ADG), average daily feed intake (ADFI), and feed conversion ratio (FCR) throughout the experiment.

ADG=(Finalweight−Initialweight)/(Daysoftest×Numberofbroilers);

ADFI=Feedconsumption/(Daysoftest×Numberofbroilers);

FCR=Feedconsumption/Weightgain;

Carcass-related traits were determined, including half-clean bore rate, full-clean bore rate, and slaughter rate. The chest and leg muscles were weighed to calculate their percentages. Villus height (VH), crypt depth (CD), and villus height/crypt depth ratio (V/C) of jejunum tissue were measured, and stained sections were observed under a Nikon Eclipse Si RS microscope (Nikon Inc., Tokyo, Japan) as described by Song et al. (2023).

To assess nutrient digestibility, three 21-day-old broilers were randomly selected from each group and tested according to the method described by Hafeez et al. (2016). The weight loss of dry matter (DM) was determined using 103∼105°C drying method, crude protein (CP) was determined by the Kjeldahl nitrogen determination method, and Ether extract (EE) was determined by the ether extraction method.

Nutrientconsumptionrates%=[Nutrientintake(g)−Nutrientexcretion(g)]/Nutrientintake(g)

### Analysis of bacterial divearsity in the intestinal tract through high throughput sequencing

After feeding the chickens for 2 weeks, three chickens were randomly selected from each pen. The intestinal contents of these chickens were mixed into 1 sample. The cecal contents of the chicken intestine were used for the follow-up experiment. Three samples were collected from each group to analyze the intestinal microbial community structure. Genomic DNA in the intestinal samples was extracted using the E.Z.N.A. Soil DNA Kit (Omega Biotek, USA). The forward primer 341 F: 5′-CCTAYGGGRBGCASCAG-3′ and reverse primer 806R: 5′-GGACTACNNGGGTATCTAAT-3′ were used to amplify the 16S rRNA gene V3+ V4 region. The 20 μL PCR mixture contained 10 ng template DNA, 4 μL 5 × FastPfu buffer, 2 μL of 2.5 mM DNTP, 1.0 U of Taq polymerase, 0.8 μL of each primer of 5 µM, 0.2 μL BSA, and distilled water. The amplification procedures were as follows: 95°C for 3 min; 27 cycles of 95°C for 30 s, 55°C for 30 s, and 72°C for 45 s; and final extension at 72°C for 10 min. Using Illumina HiSeq 2500, the PCR product was sequenced by Beijing Biomarker Technologies Co. Ltd.

### Sequence analysis

The high-quality sequences were clustered into different operational taxonomic units (OTUs) by using Qiime (V1.9.1) (Caporaso et al., 2010) software at 97% sequence similarity. The sequences were aligned by referring to the GreenGenes reference gene database. The diversity analysis of the dataset was performed in two ways: one is by measuring the alpha diversity of individual samples and the other is by evaluating beta diversity between the samples. The alpha (Grice et al., 2009) diversity analysis (the rarefaction curve, Chao1 richness, Shannon index, and Simpson diversity index) is used to estimate bacterial biodiversity in a single sample. The rarefaction curve is used to evaluate whether the produced data are sufficient to cover all species in the community. Chao1 and ACE indicate the species richness of the community. The Shannon index and Simpson reflect the abundance of each species, including species richness and species evenness. The beta diversity analysis is conducted to assess bacterial distribution and content and evaluate the total diversity in different samples based on the bacterial profile.

### Statistical analysis

All experiments were repeated three times. Data are expressed as mean ± standard deviation. IBM SPSS Statistics 21.0 software (SPSS Inc., USA) was employed to conduct one-way ANOVA and T-test (P < 0.05).

## Results

### Chemical composition analysis of FSBM

Table 2 presents the chemical composition, hygienic indicators, and ANFs of SBM and FSBM. FSBM fermented by ED-3-7 changed its chemical composition. The contents of crude protein, acid-soluble protein, and total acid in FSBM increased significantly by 11.68%, 342.61%, and 293.03%, respectively, compared with those in SBM. The contents of crude fiber, lipid, NDF, ADF, and ash significantly reduced by 33.48%, 27.28%, 8.86%, 1.73%, and 4.80%, respectively. Strain ED-3-7 reduced the pH value of FSBM from neutral (6.51) to acidic (4.68). Aflatoxin was 3.34 µg/kg in SBM, but not detected in FSBM. Both samples had <0.3 MPN/g coliforms bacteria. Compared with SBM, the contents of glycinin, β-conglycinin, urease, and phytic acid significantly decreased in FSBM by 82.04%, 88.42%, 93.10%, and 80.93%, respectively (P < 0.05). The TI content in SBM was 23.63 mg/g, whereas that in FSBM was lower than that specified as the detection limit of the ELISA test kit (0.3 mg/g).

**Table 2:** Anti-nutritional factors (ANFs), chemical composition, and hygienic indicators in SBM and FSBM.

	**SBM**	**FSBM**
Chemical composition		
CP (Crude protein), %	43.15±0.45b	48.19±0.10a
ASP (acid-soluble protein), %	2.91±0.06b	12.88±0.08a
ASP/CP, %	6.74b	26.73a
Ash, %	6.46±0.04a	6.15±0.08b
Total acid, g/kg*	2.87±0.14b	11.28±0.23a
Crude fiber, %	9.05±0.04a	6.02±0.02b
Lipid, %	1.10±0.01a	0.80±0.01b
Neutral detergent fiber, %	7.90±0.01a	7.20±0.02b
Acid detergent fiber, %	11.53±0.03a	11.33±0.02b
pH*	6.51±0.02a	4.68±0.12b
Water content (%)	9.00±0.06b	48.62±0.35a
Hygienic indicator*		
Aflatoxin, μg/kg	3.34	No detected
Coliform bacteria, MPN/g	<0.3	<0.3
ANFs (anti-nutritional factors)		
Glycinin, mg/g	88.15±0.03^a^	15.83±0.03^b^
β-conglycinin, mg/g	92.78±0.03^a^	10.74±0.05^b^
Trypsin inhibitor, mg/g	23.63±0.12	<0.3
Urease, U/g	0.29±0.01	0.02
Phytic acid, %	2.15±0.04	0.41

Note: SBM, soybean meal; FSBM, soybean meal fermented with ED-3-7; *wet basis, unlabeled as DM basis; MPN, most probable number; means in a raw with different letters were significantly different (p < 0.05).

Table 3 presents the amino acid content of SBM and FSBM. After SBM fermentation, the content of all types of amino acids increased. Among them, the contents of lysine and histidine in FSBM increased by 24.11% and 7.48% compared with those in SBM.

**Table 3:** Amino acid content in soybean meal (SBM) and fermented soybean meal (FSBM).

	**SBM**	**FSBM**
Aspartic acid, %	4.71	4.88
Threonine, %	1.68	1.73
Serine, %	2.05	2.11
Glutamic acid, %	7.52	7.83
Proline, %	2.30	2.34
Glycine, %	1.85	1.90
Alanine, %	1.94	1.99
Valine, %	2.09	2.17
Isoleucine, %	1.98	2.04
Leucine, %	3.26	3.34
Tyrosine, %	1.39	1.42
Phenylalanine, %	2.15	2.23
Histidine, %	1.07	1.15
Lysine, %	2.24	2.78
Arginine, %	2.80	2.85
TAA	39.17	40.72

Note:SBM: soybean meal; FSBM:ED-3-7 Fermented soybean meal; TAA:Total amino acid

### Bacterial identification

**Fig. 1:**
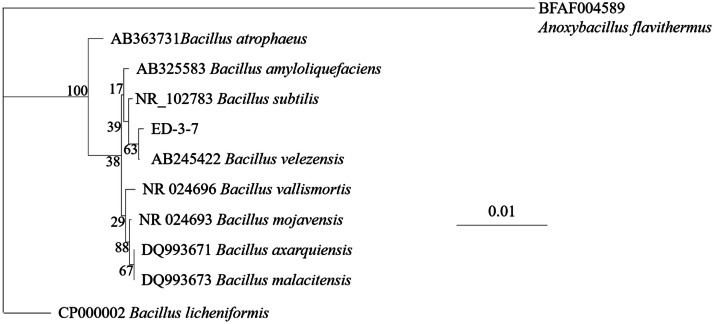
Phylogenetic tree of strain ED-3-7 based on 16S rRNA gene sequences. The phylogenetic tree was constructed by the neighbor–joining (NJ) method using the MEGA 5.1 software. The bootstrap values are shown at the branch points.

The 16S rRNA gene sequence was submitted to the GenBank database (accession number: MT950330). The strain ED-3-7 displayed approximately 99.78% similarity to *B. subtilis* 168. Fig. 1 illustrates a phylogenetic tree displaying the relationship between the strain ED-3-7 and other strains.

Biochemical and physiological characteristics indicated that strain ED-3-7 was positive for the contact enzymes test, oxidase test, Voges-Proskauer tests, nitrate reduction test, citric acid utilization, sugar alcohol fermentation, starch hydrolysis, ammonia production experiments, and lecithinase and gelatin liquefactionoxidase, whereas negative for the methyl red reaction (Table 4). Based on the results of biochemical and physiological tests and 16S rRNA gene sequences, ED-3-7 was identified as *B. subtilis*.

**Table 4:** Physiological and biochemical identifications of ED-3-7.

	ED-3-7	*Bacillus subtilis* 168
Gram stain	+	+
Growth in 2%NaCl	+	+
Growth in 5%NaCl	+	+
Growth in 7%NaCl	+	+
Growth in 10%NaCl	+	+
Contact enzymes	+	+
Oxidase test	+	+
Motility	+	+
Methyl red reaction	-	-
Voges-proskauer(VP) tests	+	+
Nitrate reduction test	+	+
Citric acid utilization	+	+
Sugar alcohol fermentation	+	+
Starch hydrolysis	+	+
Ammonia production experiments	+	+
Lecithinase	+	+
Gelatin liquefaction	+	+

Note: + positive - negative

### Effect of FSBM on broiler chicken growth performance

Table 5 shows the growth performance of SBM and FSBM-fed birds. There was no significant difference in initial body weight between the two groups, but the final body weight and ADG in FSBM group were increased significantly. After slaughter, the carcass traits improved with FSBM feeding, showing a significant increase in the full clean bore rate and no significant change in other indicators. Feeding FSBM improved the intestinal villus morphology and structure of broilers, and may have promoted the digestion and absorption of nutrients in the their intestines. Compared with feeding SBM, the apparent digestibility of crude fat and CP in broiler chickens increased by 6.71% and 5.23% after feeding FSBM.

**Table 5:** Effects of FSBM on the growth performance of White Feather Broiler chicken.

	SBM	FSBM
**Growth index**		
Initial weight,g	181.35±2.73a	181.52±2.81a
Final weight,g	948.70±6.33b	968.65±6.98a
ADFI (g/bird per day)	98.32±1.92a	99.76±2.44a
ADG (g/bird per day)	54.81±0.45b	56.22±0.30a
FCR (g/g)	1.79±0.02a	1.77±0.03a
**Carcass Traits**		
Slaughtering rate (%)	87.96±1.00a	88.87±0.91a
Half clean bore rate (%)	78.26±1.02a	79.44±1.19a
Full clean bore rate (%)	72.93±1.17b	74.02±0.99a
Chest muscle rate (%)	15.22±1.01a	16.30±1.00a
Leg muscle rate (%)	17.98±0.99a	19.06±0.79a
**Intestinal health**		
Villus height,μm	984.29±12.90b	1028.88±37.01a
Crypt depth,μm	113.71±6.60a	118.18±9.15a
Villus height/ Crypt depth	8.67±0.51a	8.74±0.47a
**Nutrient digestibility**		
Dry matter (%)	72.03±1.72b	79.27±1.35a
Ether extract (%)	86.17±0.68b	91.95±1.56a
Crude protein (%)	63.10±0.96a	66.40±1.05a

Note:SBM: soybean meal; FSBM:ED-3-7 Fermented soybean meal. ADFI: average daily feed intake; ADG: average daily gain; FCR: feed conversion ratio

### Changes in flora after FBSM feeding

Chickens were selected for a 2-week feeding experiment with SBM and FSBM. The intestinal contents were collected and sent to sequencing companies for testing so as to observe feeding-induced changes in the bacterial structure.

#### Sequencing and clustering analysis

**Fig. 2:**
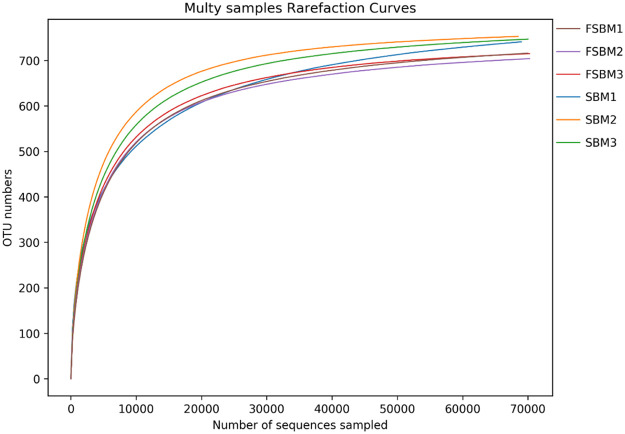
Rarefaction curves based on the observed species value. The rarefaction curve was plotted where the X-axis represents the number of clones (sequences) and the Y-axis represents the number of observed species.

Fig. 2 illustrates the rarefaction curves of intestinal content samples of SBM- and FSBM-fed chicken. The rank abundance curve indicated that the sequencing depth in intestinal contents was adequate for further analysis. The rarefaction curve of each sample increased precipitously and later approached to become flat. This indicated that the sequencing depth was sufficient to replicate the diversity of the intestinal bacterial community. The coverage rate of species was >99.8% for all samples (Table 6). This indicated that most information can be obtained from the current sequencing data. The sequencing results met the analysis requirements.

**Table 6:** Samples alpha diversity analysis index.

**Sample ID**	**Feature**	**ACE**	**Chao1**	**Simpson**	**Shannon**	**PD_whole_tree**	**Coverage**
FSBM	711.67±3.84	731.57±4.75	741.26±7.56	0.957±0.003	6.29±0.81	45.27±0.42	0.9993±0.00
SBM	747.00±3.46	769.89±6.53	782.11±9.49	0.974±0.006	6.81±0.18	47.49±0.06	0.9992±0.00
P-value	0.02	0.009	0.28	0.60	0.059	0.006	0.67

Note:SBM, soybean meal; FSBM, soybean meal fermented with ED-3-7.

#### Bacterial alpha diversity

The alpha diversity metrics summarize an ecological community's structure with respect to its richness, evenness, or both (Willis, 2019). It is used for analyzing microbial community diversity within the community (McKie and MccleAry, 2016), including ACE, Shannon, Simpson, PD value, Chao1, and commodity coverage. Shannon, Simpson, and PD values reflect population diversity, while ACE and Chao1 reflect population richness. Table 6 presents the alpha diversity index of the sample. The Shannon, Simpson, and Chao1 indices and PD values of the SBM group were larger than those of the FSBM group. This indicated that the bacterial abundance and diversity of the SBM group were higher than those of the FSBM group. The feature value also confirmed the presence of more microbial in SBM than in FSBM.

#### out-specific analysis

**Fig. 3:**
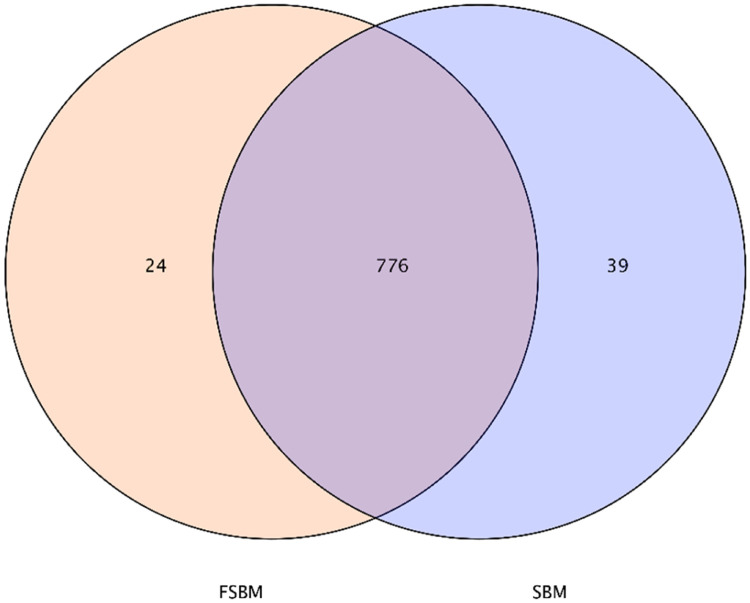
Venn diagram analysis based on operational taxonomic units (OTUs) for different groups. The number of overlapping portions represents the number of OTUs shared between the two samples. The portion that does not overlap represents the amount of OUT that is specific to the sample.

The Venn diagram in Fig. 3 clearly reflects the number of OTUs between groups. It also presents the degree of similarity and specificity between groups. In total, 839 OTUs were present in both groups. The FSBM group had 24 OTUs of specific bacteria, which accounted for 2.8%, while the SBM group had 39 OTUs of specific bacteria, which accounted for 4.6%. In total, 776 OTUs of the same bacteria were present in the two groups, which accounted for 92.5%. Feeding FSBM changed the microbial composition in the chickens’ intestinal contents, which is consistent with the results of the Feature present in Table 6.

#### Taxonomic unit-level analysis of species

**Fig. 4:**
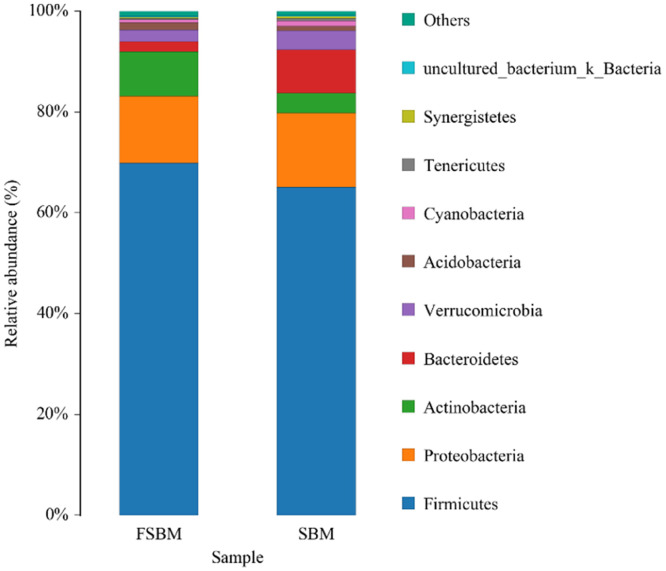
Relative abundance of species at the phylum level. The abundance is represented in terms of the percentage of the total effective bacterial sequences in the sample. The top 11 abound taxa are displayed.

Species can be categorized into five taxonomic units: phylum, class, order, family, and genus. Fig. 4 displays species with abundant samples and their proportion at the phylum level. The top 9 dominant phyla in the 6 samples were Firmicutes, Proteobacteria, Actinobacteria, Bacteroidetes, Verrucomicrobia, Acidobacteria, Cyanobacteria, Tenericutes, and Synergistetes, and the proportion of all phyla changed in the samples. In the SBM group, Firmiculosis was the dominant bacterial community, which accounted for 65.1%. The proportion of Firmiculosis in the FSBM group increased to 69.9%. The proportion of Proteobacteria and Bacteroidetes decreased from 14.7% and 8.7% in the SBM group to 13.2% and 2.0% in the FBSM group, respectively. The proportion of Actinobacteria increased from 3.9% in the SBM group to 8.8% in the FBSM group. In the other phyla, apart from the increase in the abundance of Acidobacteria in the FSBM group compared with the SBM group, all other phyla exhibited a decrease in abundance.

**Fig. 5:**
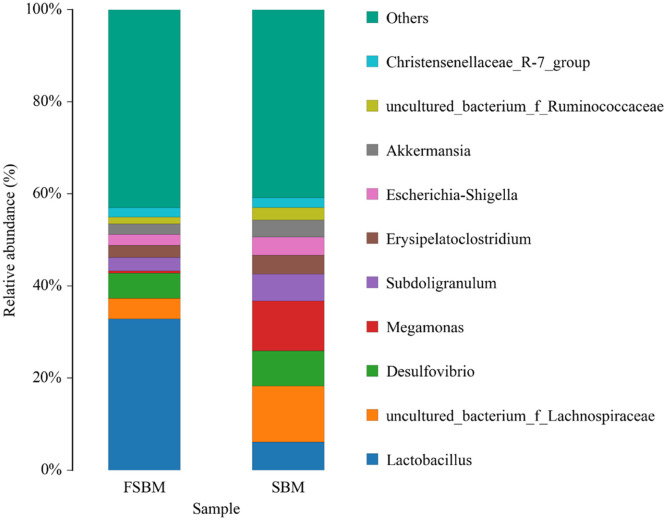
Relative abundance of the genus-level species. Abundance is expressed as a percentage of the total active bacterial sequence in the sample. The top 11 enrichment groups are shown.

**Fig. 6:**
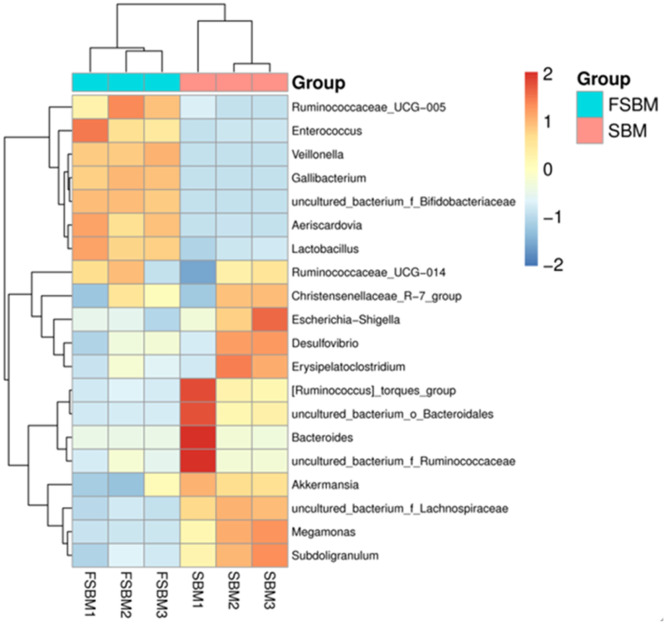
A horizontal heat map of the genus level. The horizontal heat map of the genus level, in which each column represents a sample, each row represents a species, and the colors in the Fig. represent the relative abundance of species in the group of samples. For the specific trend of abundance size, please see the numerical label under the color bar on the upper right-side. On the left side is the tree diagram of species clustering, and on the right side is the name of the species. The smaller the distance between two species branches, the closer they are.

*Lactobacillus, uncultured_bacterium_f_Lachnospiraceae, Desulfovibrio, Megamonas, Subdoligranulum, Erysipelatoclostridium, Escherichia-Shigella, Akkermansia, uncultured_bacterium_f_Ruminococcaceae,* and *Christensenellaceae*_*R-7_group* were the main genera in the chickens’ intestinal contents (Fig. 5). The dominant genus in the SBM group was *uncultured_bacterium_f_Lachnospiraceae* and *Megamonas*, whereas that in the FSBM group was *Lactobacillus*, which accounted for 32.9%. The heatmap at the genus level presented the top 20 species with high abundance in the SBM and FSBM groups (Fig. 6). Abundance was indicated by the shade of a color. Compared with the SBM-fed chickens, visualization exhibited significant genus-level differences in the gut microbiota of the FSBM-fed chickens.

#### Analysis of change in the bacterial community between groups

**Fig. 7:**
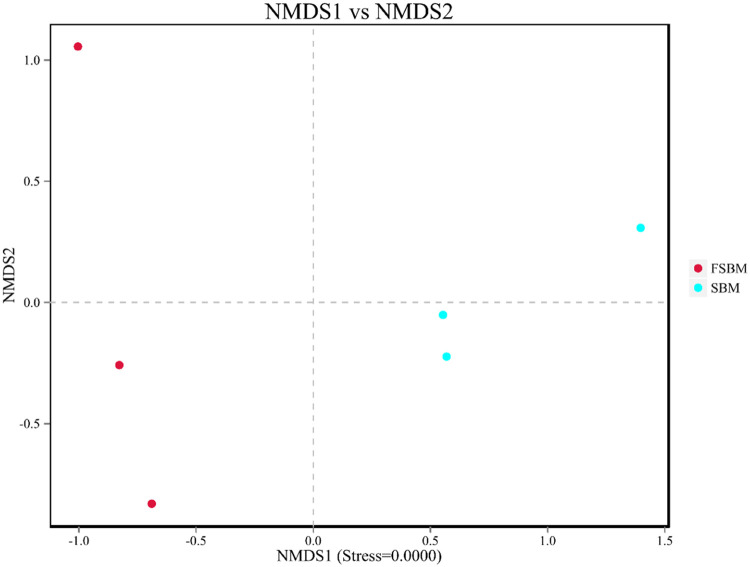
Non-metric multi-dimensional scaling plot based on weighted UniFrac distance. Points with the same pattern represent the three replicate samples from the same group.

In nonmetric multidimensional scaling analysis, the degree of difference between samples is determined on the basis of the distance between points (Fig. 7). The difference between groups was greater than that within groups, which indicates that grouping is meaningful. The bacterial community of the FSBM group was significantly distinct from that of the SBM group. The distance between the three points in the Fig. was larger. This observation suggests that a greater difference existed between the three groups and that the bacterial community of the FSBM group underwent significant changes, whereas that of the SBM group underwent minor changes.

## Discussion

### *SBM fermented by* Bacillus

SBM, as a feed, is a crucial raw material and source of plant protein. It is the most common source of dietary protein in poultry farming. However, the macromolecular protein and ANFs contained in SBM reduce the SBM nutritional value (Lee et al., 2022) and affect its absorption by animals (Dai et al., 2022; Feng et al., 2007; Yang et al., 2018). Glycinin, β-conglycinin, phytic acid, and TI are the main ANFs in SBM (Adeyemo and Onilude, 2013; Yan et al., 2022). Microbial fermentation is among the effective solutions (Wang et al., 2020). Microbial fermentation not only degrades macromolecular proteins and ANFs but also improves the SBM's nutritional value (Shi et al., 2017b) and palatability. *B. subtilis* is among the most commonly used strains in SBM fermentation. *B. subtilis* is widely used for various aspects and its safety is recognized (Kimura and Yokoyama, 2019). It can regulate the balance of intestinal microbiota, improve animal immunity, and promote animal growth and development (Abudabos et al., 2020). Studies have assessed the quality of SBM fermented by *B. subtilis*. Moreover, the macromolecular proteins in SBM are degraded into small molecular polypeptides, and *B. subtilis* exhibits a strong ability to degrade ANFs, so that the application value of SBM is improved (Kim et al., 2015; Teng et al., 2012). *B. subtilis* is also a common probiotic.

The quality of FSBM is attributable to the degradation of macromolecular proteins and ANFs by various microbial enzymes, and protease is the key enzyme. The amount and type of protease may relate to the FSBM characteristics (Qi *et al.*, 2023). *B. subtilis* produces some metabolites such as proteases and other hydrolases. These can hydrolyze proteins and other macromolecular substances into peptides, glucose, etc., thereby improving the SBM's nutritional value (Feng *et al.*, 2007; Qi *et al.*, 2023). Furthermore, *B. subtilis* in the animal intestine can inhibit the reproduction of harmful bacteria, thereby improving the animal's resistance (Abudabos *et al.*, 2020). The *Bacillus-*produced neutral protease may play a role at the beginning of fermentation, and then the acid protease gradually becomes active with a decrease in pH (Qi *et al.*, 2023). In this study, ED-3-7 produces high levels of neutral and acid proteases, which may be the reason why this strain can improve the SBM quality. In addition, the post-inoculation decrease in the pH value of FSBM may be related to acetic acid produced by the dominant bacteria *Bacillus* and *Lactobacillus* during fermentation (Cui et al., 2020).

According to the current studies, aerobic solid fermentation is the main method of *Bacillus*-mediated fermentation. On assessing the effect of *B. siamensis* JL8 on SBM through aerobic solid-state fermentation, Zheng et al. found that the contents of ANF, β-conglycinin, and TI were significantly reduced by 86.0%, 70.3%, and 95.01%, respectively (Zheng *et al.*, 2017). *Bacillus* is a facultative anaerobic bacterium that can grow under anaerobic fermentation conditions. This indicates that *Bacillus* can ferment SBM through anaerobic solid-state fermentation. Currently, *Bacillus* species are used alone or in combination with other microbes for the anaerobic fermentation of SBM and cottonseed meal (Li et al., 2022)^.^ Anaerobic solid-state fermentation avoids the disadvantages of aerobic fermentation, such as susceptibility to fungal infections, high energy consumption, high costs, and large space requirements.

### FSBM is beneficial for chicken growth

SBM has various ANFs, which can interfere with nutrient digestion and absorption in animals and negatively affect animal health (Shi *et al.*, 2017a). Glycinin and β-conglycinin are the main ANFs in SBM. They cause the absorption disorder of livestock and poultry after they are fed, which leads to an abnormal immune system, allergic reactions in animals, and damage to their intestinal tract (Wang et al., 2014; Zhu et al., 2017). Microbial fermentation augments the SBM nutritional quality by reducing ANFs and degrading macromolecular proteins. It also provides beneficial probiotics for animals, thereby reducing SBM adverse effect on nutrient absorption in chickens and promoting their growth (Irawan et al., 2022). Feeding the fermented SBM to piglets and other young animals can reduce the feed-to-gain ratio in the animals, improve diarrheal symptoms, and enhance the performance of the animals (Zhu *et al.*, 2017). Adding FSBM to chicken feed significantly improves production performance and gastrointestinal health (Li *et al.*, 2020). FSBM added to the diet increases feed utilization, nutrient digestibility, and amino acid transport capacity of chickens (Irawan *et al.*, 2022), increasing their body weights (BWs) and muscle nutritional values. The average weight gain, final weight, digestive enzyme activity, and feed efficiency of the chickens also improved significantly(Jazi *et al.*, 2018; Jazi et al., 2019; Li et al., 2019a). In this study, feeding FSBM resulted in a significant increase in final weight, ADG, nutrient digestibility, which may be due to changes in the intestinal villi morphological structure that promoted the digestion and absorption of nutrients in broilers. This experiment further confirmed that FSBM positively affects the growth of chickens.

### FSBM changed the intestinal bacterial community

The intestinal flora greatly influences the maintenance of the normal physiological structure and function of the intestine. The growth performance of animals is closely related to their gut flora (Chen and Yu, 2020; Li *et al.*, 2019a). Diet is among the main factors affecting the gut microbiome composition(Diaz Carrasco et al., 2019). Changing the diet composition may rapidly affect the gut microbiome composition (Guo et al., 2018). According to relevant studies, FSBM can inhibit the colonization of *Salmonella* pathogens and improve the immune status and intestinal morphology (Jazi *et al.*, 2019).

We here used high-throughput sequencing technology to analyze the effect of FSBM on the intestinal microbial community of the chickens. The abundance and diversity of the intestinal flora decreased after FSBM feeding. At the dominant phyla level, Firmicutes and Proteobacteria dominated, and these two bacterial types are the most common in the environment (Rastogi et al., 2013). By using FSBM partially or completely instead of SBM in chicken diets, the relative abundance of undesirable bacteria in Proteobacteria can be reduced (Jazi *et al.*, 2018; Li *et al.*, 2022), which is consistent with this study. Adding different probiotics to the broiler feed can reduce pathogenic strains and increase intestinal Firmicutes (Abdel-Raheem et al., 2023). In this study, the abundance of Firmicutes also increased after FSBM feeding. In the gut, Firmicutes are involved in the breakdown and energy utilization of polysaccharides because their genes encode non-starch polysaccharide-degrading enzymes (Crisol-Martínez et al., 2017; Singh et al., 2012). The increased proportion of Firmicutes is a good signal for enhancing the activity of non-starch carbohydrate-degrading enzymes and is positively correlated with the improvement in animal production performance (Crisol-Martínez *et al.*, 2017; Irawan *et al.*, 2022). This could explain the increase in the final BW of chickens in the FSBM group.

The dominant genus of FSBM was *Lactobacillus*, which was 5 times more abundant than that on SBM. The abundance of lactic acid bacteria increases after fermentation, and so, FSBM can be used as a probiotic source in chicken feed (Guo *et al.*, 2018). FSBM can promote the colonization of lactic acid bacteria in the chicken cecum, thereby augmenting intestinal integrity (Kumar et al., 2022). Lactic acid bacteria exert beneficial effects on the gastrointestinal tract and chicken growth and are among the common probiotics in animal production (Ma et al., 2018; Wang et al., 2019). *Lactobacillus* promotes animal growth by protecting the gut from pathogens and increasing the rate of nutrient and energy extraction (Wang *et al.*, 2019).

## Conclusion

*B. subtilis* ED-3-7 effectively degraded macromolecular proteins and ANFs in SBM through anaerobic solid fermentation, improved the SBM quality, and was conducive to nutrient digestion and absorption in chickens. After the chickens were fed FSBM, their intestinal flora changed. The increase in the abundance of lactic acid bacteria is beneficial for the intestinal health of the chickens. The increase in the final weight proves that FSBM has a beneficial promoting effect on chicken growth, which may be related to the positive changes in their intestinal tract after being fed with FSBM.

## Declaration of competing interest

The authors declare the following financial interests/personal relationships which may be considered as potential competing interests:

Tongguo Gao reports financial support was provided by Hebei Agricultural University. If there are other authors, they declare that they have no known competing financial interests or personal relationships that could have appeared to influence the work reported in this paper.
